# A telomere-to-telomere genome assembly of *Nymphaea minuta* provides details into the developmental transcriptome atlas and adaptive regulatory mechanisms

**DOI:** 10.1093/hr/uhag085

**Published:** 2026-03-04

**Authors:** Hongliang Chen, Yufan Liang, Jia-Yu Xue, Fei Chen

**Affiliations:** National Key Laboratory for Tropical Crop Breeding, Sanya Institute of Breeding and Multiplication, College of Tropical Agriculture and Forestry, Hainan University, Sanya 572025, China; National Key Laboratory for Tropical Crop Breeding, Sanya Institute of Breeding and Multiplication, College of Tropical Agriculture and Forestry, Hainan University, Sanya 572025, China; College of Horticulture, Bioinformatics Center, Academy for Advanced Interdisciplinary Studies, Nanjing Agricultural University, Nanjing 210095, China; National Key Laboratory for Tropical Crop Breeding, Sanya Institute of Breeding and Multiplication, College of Tropical Agriculture and Forestry, Hainan University, Sanya 572025, China

## Abstract

The evolutionary history of the ANA-grade angiosperms provides a crucial window into the transition of early flowering plants. Within this group, the Nymphaeales (water lilies) are pivotal, yet a lack of gapless genomic resources has hindered research into their complex developmental and adaptive programs. In this study, we present a telomere-to-telomere (T2T), gap-free genome assembly of *Nymphaea minuta*, a miniature water lily endemic to Madagascar. Utilizing PacBio Revio HiFi and Hi-C technologies, we generated a 382-Mb assembly anchored to 14 chromosomes. Comparative analysis reveals a compact genome with lower levels of ancient polyploidization than other Nymphaeaceae. By integrating a comprehensive transcriptome atlas of 15 organs and developmental stages, we identified seven primary developmental trajectories and 1179 organ-specific genes. Our analysis uncovered two critical regulatory models: Sequential Dual-Module Relay: In leaves, water fluctuation triggers an initial MAPK-signaling stress response, followed by a post-transcriptional ‘transcriptome reset’ mediated by the RNA degradation pathway (LSM1/2 and ENOC) during severe drought. Energy-Program Coordination: Seed development is governed by a three-phase transition where the glyoxylate cycle (MLS) drives energy mobilization, while an ERF1-centered hub integrates ethylene, ABA, and JA signaling to balance rapid germination with immune defense. These findings provide a definitive genomic reference for basal angiosperms and elucidate the molecular networks enabling the survival and rapid development of these ancient aquatic herbs.

## Introduction

The sudden and explosive radiation of angiosperms during the Cretaceous period remains one of the most enigmatic events in evolutionary history, a phenomenon Charles Darwin famously termed an ‘abominable mystery’ in a letter to Joseph Hooker in 1879 [1]. This ‘mystery’ encompasses not only the origin of flowering plants but also the rapid development of specialized floral characters and the regulatory networks that allowed them to colonize nearly every terrestrial and aquatic ecosystem on Earth [1]. Modern phylogenomics has provided clarity by identifying the ‘ANA-grade,’ Amborellales, Nymphaeales, and Austrobaileyales, as the lineages that diverged earliest from the line leading to the mesangiosperms, which contain the vast majority of extant flowering plants [2]. Among these basal lineages, the order Nymphaeales, commonly known as water lilies, occupies a critical evolutionary space [3]. While *Amborella trichopoda* is a singular, dioecious shrub with simple flowers restricted to New Caledonia, the Nymphaeales are globally distributed, hermaphroditic, and possess a diverse array of showy floral colors and scents that attract varied pollinators [3, 4]. These traits make them superior models for investigating the evolution of characters that defined the success of mesangiosperms, such as floral scents, vibrant pigmentation, and synchronized reproductive cycles. However, research into the Nymphaeales has been historically constrained by the lack of genomic resources. Most species exhibit high ploidy levels, large genomes, and extensive ancient polyploidy events, making high-quality assemblies difficult to achieve [5].

In the current genomic era, the pursuit of telomere-to-telomere (T2T) assemblies has become the gold standard for plant research [6, 7]. Traditional ‘draft’ genomes, even those at the chromosome scale, such as the *Nymphaea colorata* genome that was published by us in *Nature* [2], often contain hundreds of gaps in highly repetitive regions, particularly at the centromeres and telomeres [8]. These gaps often harbor transcriptionally active regions, structural variations, and regulatory elements that are crucial for understanding evolutionary adaptation and genome stability [9, 10]. For horticultural plants, where complex traits like color, scent, and stress resilience are often linked to repetitive regions and gene duplications, T2T genomes provide an unprecedented resolution for functional genomics [10].

Beyond the architecture of the genome, the developmental plasticity of water lilies is governed by sophisticated transcriptomic programs that integrate internal developmental cues with external environmental signals [3]. Living in aquatic-terrestrial interfaces, these plants must navigate extreme physiological transitions, such as the move from submerged hypoxia to emergent dehydration [11]. While Mitogen-Activated Protein Kinase (MAPK) cascades are well-known transducers of abiotic stress in higher plants [12], the role of post-transcriptional mechanisms, is only beginning to be explored in basal angiosperms [13]. Similarly, the transition from heterotrophic seed germination to autotrophic seedling establishment involves massive metabolic reprogramming, necessitating the activation of the glyoxylate cycle to mobilize lipid reserves [14]. The integration of these pathways by master regulators is a window into how early flowering plants balanced growth and defense.

In this study, we report the first T2T genome assembly of *Nymphaea minuta*. By leveraging high-fidelity long-read sequencing and Hi-C data, we provide a gapless reference for this critical evolutionary lineage. We further construct an exhaustive developmental transcriptome atlas covering 15 organs and multiple time points for germination and stress response. Our findings reveal the molecular relays that coordinate leaf resilience and seed establishment, establishing *N. minuta* as a powerful laboratory model for elucidating the early evolution and adaptive strategies of the angiosperms.

## Results

### Phenotype of *N. minuta*

**Figure 1:**
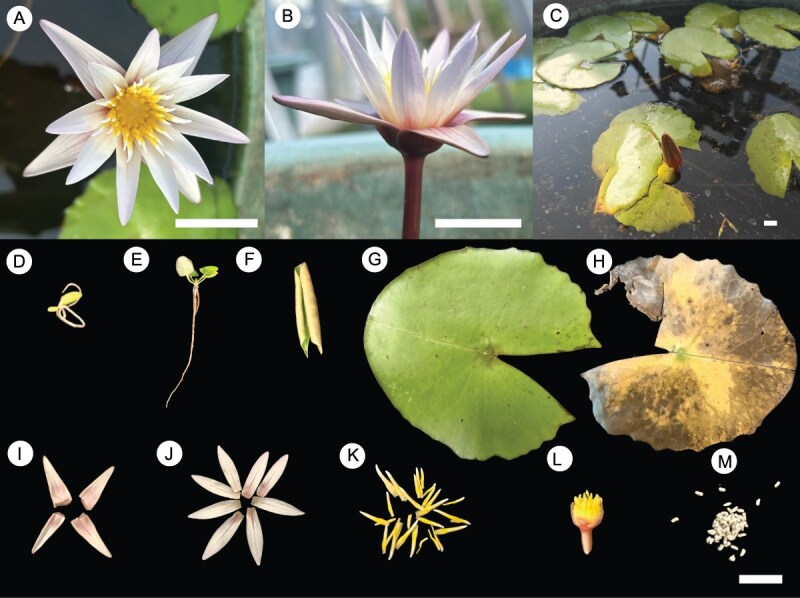
Morphological characteristics of *N. minuta*. (A) Top view of a fully opened flower showing the arrangement of petals and the central yellow stamens. (B) Side view of the flower illustrating the receptacle and the transition from sepals to petals. (C) Natural growth habit in an aquatic environment, displaying floating leaves and a developing floral bud. (D) Germinating seed with emerging radicle. (E) A complete young plantlet with developing roots and submerged leaves. (F) The first true leaf. (G) A mature, healthy floating leaf with a characteristic basal sinus. (H) A senescing leaf. (I) Sepals; (J) outer petals; (K) stamens; (L) carpel; and (M) dry seeds. Scale bar = 1 cm.

*Nymphaea minuta* (homonym *N. dimorpha* I.M. Turner or *N. nikitinii* Doweld) has emerged as a promising candidate for a model species within the water lily order. Endemic to the canopy-shaded rain pools of coastal Madagascar, this species is a miniature, diploid water lily characterized by remarkable adaptability and a rapid life cycle [15]. Unlike the previously proposed model *N. thermarum*, which is critically endangered and requires highly specialized growth conditions such as thermal mud and high CO_2_ levels to flower, *N. minuta* is far easier to cultivate in laboratory settings. It produces a large number of seeds per flower, exhibits year-round blooming, and can propagate asexually through tubers, providing versatile options for genetic and developmental studies. Moreover, *N. minuta* displays phenotypic plasticity through two distinct growth forms: a submerged form with thin, flaccid leaves and cleistogamous flowers (Fig. 1), and an emergent form with floating leaves and showy, chasmogamous flowers [16].

### Telomere-to-telomere reference genome of *N. minuta*

The genome of *N. minuta* was assembled using a hybrid approach combining PacBio Revio HiFi long-read sequencing and Hi-C chromatin configuration capture. We extracted high-molecular-weight DNA from young leaves of plants grown under controlled conditions in Sanya, Hainan. HiFi sequencing yielded 56.93 Gb of 7.89 million reads with an N50 of 15 679 bp and a median quality value (QV) of 37, while Hi-C sequencing provided 50.13 Gb of 215 million valid read pairs. The initial assembly using hifiasm was scaffolded into 14 chromosomes, followed by gap filling and single-base polishing.

The final assembly reached 382 Mb in size, which is notably more compact than the draft genome of *N. colorata* (409 Mb, 2*n* = 28) and *N. thermarum* (498.78 Mb, 2*n* = 28) [17] (Table S1). This compactness is primarily attributed to a lower density of repetitive elements and ancient polyploidization remnants. Chromosome integrity was validated through the identification of 27 telomeres (Table S2) and 14 centromeres (Table S3) across the 14 chromosomes using the quartet pipeline. All chromosomes were gap-free, representing the highest-quality assembly for any species in the Nymphaeales to date (Table 1).

**Table 1:** Comparative genomic statistics of *N. minuta* against related species within Nymphaeaceae.

**Species**	**Genome size (Mb)**	**Assembly quality**	**Chromosomes (n)**	**No. of genes**
*Nymphaea minuta*	382	T2T (Gap-free)	14 (2*n* = 28)	21 860
*Nymphaea colorata*	409	Chromosome-scale	14 (2*n* = 28)	31 580
*Nymphaea thermarum*	498.78	Draft	14 (2*n* = 28)	25 760
*Nymphaea mexicana*	~500	Chromosome-scale	14 (28*n* = 56)	56 194
*Victoria amazonica*	4557	Chromosome-scale	12 (2*n* = 24)	50 310

**Figure 2:**
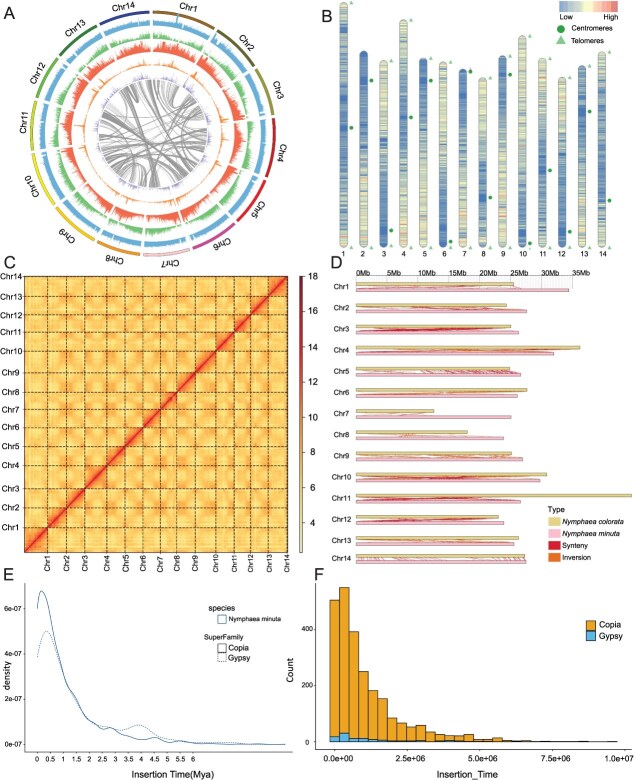
Characteristics of the *N. minuta* T2T genome assembly and comparative genomics. (A) Circos plot illustrating the genomic features across the 14 chromosomes. The tracks (from outer to inner) represent: (i) chromosome ideograms (Chr1–Chr14) measured in Mb; (ii) gene density; (iii) repeat sequence density; (iv) GC content; and (v) syntenic blocks represented by internal links. All densities were calculated in 100-kb windows. (B) Landscape of chromosome features. Heatmaps show the distribution of genomic elements (likely gene or repeat density) along each chromosome. Triangles and circles denote the identified telomeric repeats and centromeric regions, respectively, confirming the T2T nature of the assembly. (C) Hi-C contact map. The heatmap shows the frequency of long-range chromatin interactions. The 14 distinct blocks along the diagonal correspond to the 14 pseudo-chromosomes, indicating a high-quality physical map and accurate scaffolding. (D) Synteny analysis between *N. minuta* and *N. colorata*. (E) Insertion time distribution of LTR retrotransposons. The density plot shows the estimated insertion times (Million years ago, Mya) for *Copia* (solid line) and Gypsy (dotted line) superfamilies in the *N. minuta* genome, reflecting a recent burst of TE activity. (F) Detailed insertion frequency of LTR-RTs. Histogram showing the count of Copia and *Gypsy* elements based on their insertion age, further illustrating the dominant role of *Copia* elements in recent genome expansion.

Genome annotation using BRAKER3, integrated with transcriptomic evidence, identified a comprehensive set (21860) of protein-coding genes. Repeat analysis using EDTA revealed that LTR retrotransposons (*Gypsy* and *Copia*) were the dominant repetitive elements, particularly enriched in the centromeric regions. The identification of gap-free centromeres allows for the characterization of tandem repeats like *Cen-42* and specific LTR invasions that characterize nymphaealean genome evolution (Fig. 2).

### Global transcriptomic landscape and developmental trajectories

To capture the transcriptional diversity of *N. minuta*, we performed RNA-seq on 165 samples encompassing 15 distinct organs and various developmental series (Table S4). Dimensionality reduction using UMAP and hierarchical clustering revealed seven primary developmental trajectories: Architecture (petioles, pedicels), Blade (leaves), Flower (sepals, petals, stamens, pistils), Fruit, Underground (roots, tubers), Seed, and Seedling (Table S5).

The transcriptomic atlas highlighted the high degree of functional specialization in this basal angiosperm. The spatial separation of the ‘Seed’ trajectory in the UMAP plot indicated a unique transcriptional program, likely driven by the transition between dormancy and germination. In contrast, reproductive ‘hub’ organs like the fruit and pedicel clustered centrally, reflecting their role in mediating nutrient and hormonal flow between maternal tissues and developing seeds. We also observed significant proximity between Blade and Architecture trajectories, suggesting coordinated transcriptomic control of photosynthetic organs and their structural supports.

### Identification of organ-specific genes and functional modules

**Figure 3:**
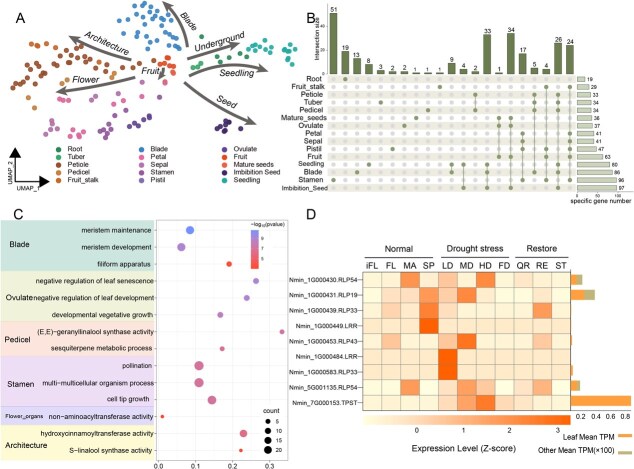
The spatiotemporal transcriptome landscape and organ-specific regulatory modules of *N. minuta*. (A) UMAP-based visualization of the *N. minuta* developmental atlas. A total of 165 transcriptome samples (including three biological replicates) are projected into a two-dimensional space using UMAP. The samples cluster into seven distinct developmental trajectories indicated by arrows: Architecture, Blade, Underground, Seedling, Seed, Flower, and Fruit. Colors represent different organs or developmental stages. (B) Distribution of organ-specific and shared expressed genes. The UpSet plot displays the number of genes specifically expressed in 15 representative organs. The horizontal bars on the right indicate the total number of specific genes identified for each organ (e.g., 96 for stamen, 86 for blade). The vertical bars represent the size of intersections (shared or unique genes) across different organ sets. (C) Functional enrichment of organ-specific and modular genes. Dot plot showing Gene Ontology (GO) enrichment analysis for specific gene sets. The *x*-axis represents the Gene Ratio, bubble size indicates gene count, and color indicates the significance level. Key enriched pathways include meristem maintenance in blades, sesquiterpene metabolic processes in the pedicel, and pollen tube tip growth in stamens. (D) Expression profiles of the leaf front-end sensing module across developmental and stress stages. Heatmap illustrating the *Z*-score normalized expression levels of core genes, including LRR-RLPs and TPST. The samples cover normal growth (iFL: immature floating leaf, FL: floating leaf, MA: mature leaf, SP: senescent leaf), drought stress (LD: light drought, MD: moderate drought, HD: heavy drought, FD: fast dry, 1h after water removal), and recovery phases (QR: quick rehydrate, 1h rehydrate, RE: recovery, ST: stable period). Bar plots on the right show the mean TPM values in leaf tissues versus other organs.

We identified 1179 organ-specific expression genes using a strict 10-fold enrichment threshold (Fig. 3). The distribution of these genes across organs revealed the molecular basis for aquatic adaptation and floral evolution. Stamens contain 474 expression specific genes. The stamen exhibited the highest degree of transcriptomic complexity, accounting for over 50% of single-organ specific genes. GO enrichment analysis showed these genes are involved in (*E*,*E*)-farnesyl linalool synthase activity and sesquiterpene metabolism, pointing to their role in synthesizing volatile organic compounds to attract pollinators. Leaves/Blade contain 158 specific expression genes. Enriched in meristem maintenance and negative regulation of leaf senescence, ensuring sustained photosynthetic capacity. Roots and Tubers are Underground Module and they contain 47 specific expression genes. These genes are involved in hypoxia adaptation and anaerobic metabolism, critical for survival in water-saturated substrates. Structural Organs form the Stem Support Module and they contain 41 specific expression genes. Enriched in pathways related to mechanical support and vascular differentiation.

Interestingly, we discovered a functional cross-regulation where ovule-specific genes were enriched in negative regulators of leaf development. This suggests a systemic evolutionary strategy where the initiation of reproductive development triggers signals that suppress vegetative growth to prioritize resource allocation to the next generation.

### A sensing module coupling senescence and early drought warning

**Figure 4:**
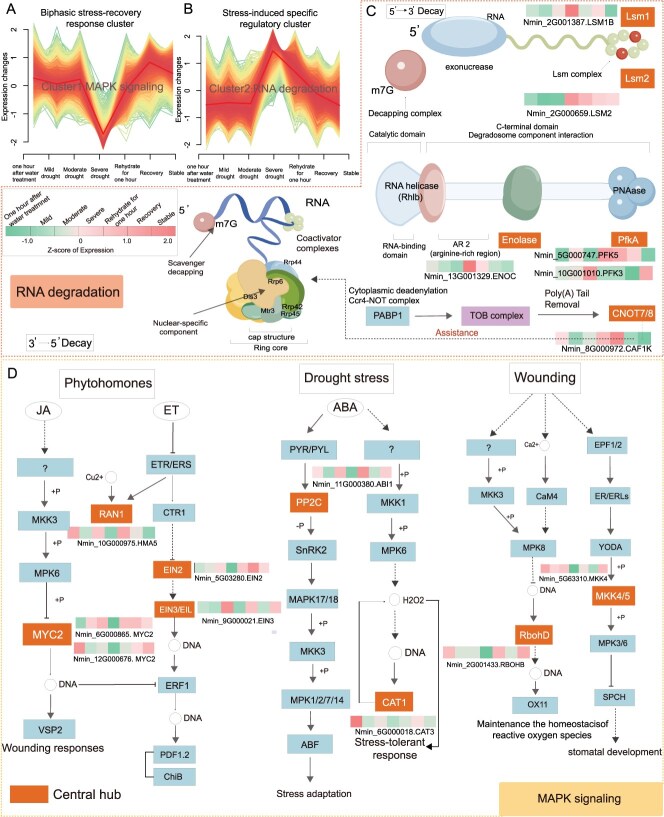
Transcriptomic landscape and adaptive regulatory mechanisms of *N. minuta* under drought stress. (A) Expression profiles of key gene clusters during drought and recovery. (B) Schematic representation of the RNA degradation machinery in *N. minuta*. (C) Integrated MAPK signaling networks in response to phytohormones and abiotic stresses. The pathways illustrate how *N. minuta* integrates various signals: Phytohormones (JA and ET): Signal transduction leading to wounding responses via the MYC2 and EIN3/EIL nodes. Drought Stress (ABA): The core PYL-PP2C-SnRK2 cascade and its downstream MAPK modules (MKK3-MPK6) leading to stress adaptation. Wounding: Rapid signaling involving Ca^2+^ flux, ROS production (RboHD), and stomatal development regulation (EPF1/2-YODA). Key Regulatory Nodes: Proteins highlighted (e.g., RAN1, EIN2, CAT1, MKK4/5) represent central hubs with significant expression changes. Heatmaps with gene IDs (e.g., Nmin_10G000975) provide specific expression data for the corresponding orthologs in *N. minuta*.

Within the leaf blade, we identified a core set of genes that function as a front-end environmental and developmental sensor. This module consists of nine genes encoding Leucine-Rich Repeat (LRR) Receptor-Like Proteins (RLPs) and a tyrosylprotein sulfotransferase (TPST, Nmin_7G000153) (Fig. 4).

These genes exhibited a precise spatio-temporal expression pattern: they were specifically upregulated during the natural senescence of leaves and during the very earliest stages of water loss (1 h post-removal from water). Their expression did not peak at the severe drought stage but rather served as a ‘first responder,’ returning to baseline immediately upon rewatering. This suggests that *N. minuta* leaf cells utilize a common surface signal hub, likely sensing cell wall changes or apoplastic pressure, to integrate developmental senescence cues with early environmental stress signals, allowing for pre-emptive physiological adjustments. Notably, this LRP-TPST cell-surface hub likely functions as a ‘primary sentinel’ that perceives initial apoplastic pressure changes or cell-wall modifications to trigger a phosphorylation cascade, which serves as the upstream activator for the Phase I MAPK-signaling network to initiate systemic adaptive responses.

### Sequential dual-module relay for leaf response to water fluctuation

The ability of aquatic plants to survive transient drying of their habitats is a major evolutionary innovation. In *N. minuta*, we identified a ‘Sequential Dual-Module Relay’ mechanism that governs the transition between growth and extreme survival modes during water fluctuations.

#### Phase I: MAPK signaling and early perception

During the initial response to water loss and the subsequent recovery phase, Cluster A genes (1066 genes) are dominant. These genes are significantly enriched in the MAPK signaling pathway, including core transcription factors like MYC2 (integrating JA signals), EIN2/3 (ethylene signals), and the ROS-generating enzyme RBOHB. This module functions as the perception engine, initiating antioxidant responses and osmotic adjustments to survive mild stress and rebuild cellular integrity during rewatering.

#### Phase II: RNA degradation and transcriptome emergency remodeling

As drought stress intensified, a tactical shift from global signal amplification to transcriptomic reset was observed, where the induction of the RNA degradation pathway likely serves to clear non-essential mRNAs, thereby prioritizing resources for survival and facilitating a rapid cellular transition into the Phase II steady state. As stress progresses to the severe drought threshold, Cluster A is suppressed, and Cluster B (890 genes) is strictly induced. Cluster B is significantly enriched in the RNA degradation pathway, encoding core components of the RNA decapping machinery, such as LSM1B, LSM2, and the exoribonuclease ENOC. This induction marks an ‘emergency reset’ of the transcriptome. By activating the LSM1-7 complex to selectively decap and degrade the existing pool of mRNA associated with normal growth, the plant can rapidly halt non-essential processes. This mechanism preserves limited water and energy for survival-critical transcripts (such as heat shock proteins and LEA genes) and creates a ‘blank slate’ for the rapid re-initiation of developmental programs once water becomes available again.

### The developmental transcriptomic map of seed germination

**Figure 5:**
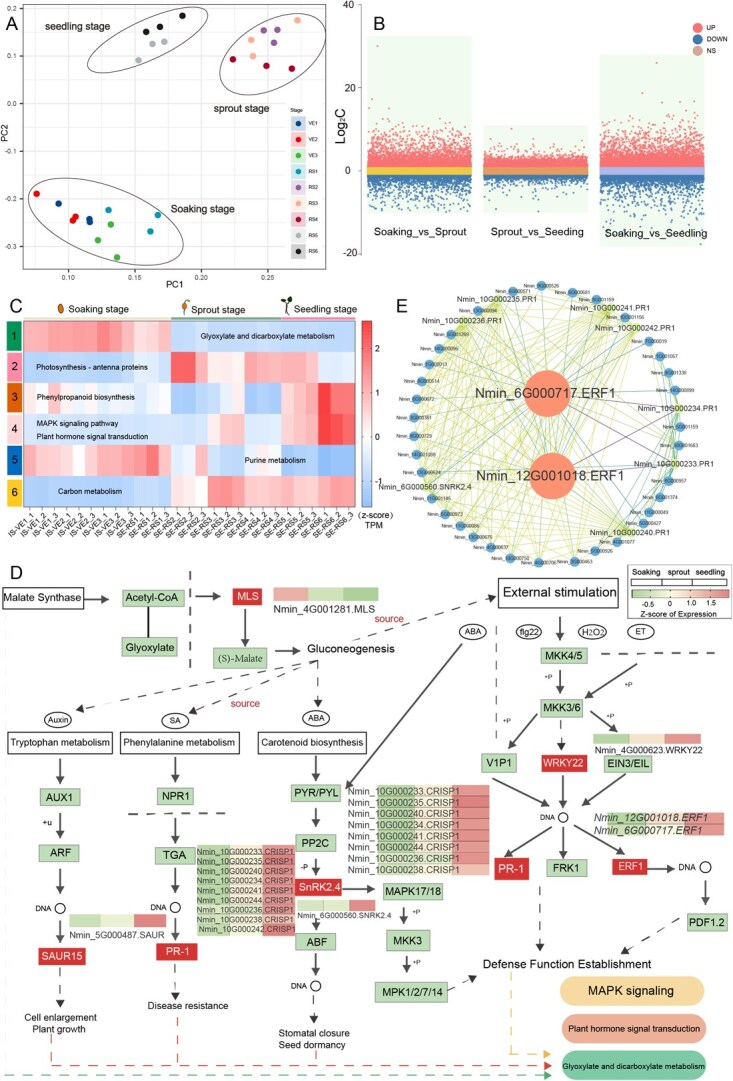
Transcriptomic dynamics and regulatory networks during *N. minuta* seed germination. (A) Principal Component Analysis (PCA) of global expression patterns, dividing the germination process into three distinct phases: Soaking, Sprout, and Seedling. (B) Differential gene expression analysis capturing the massive transcriptomic shift across developmental transitions. (C) K-means clustering of dynamic genes into distinct expression modules, highlighting key biological processes such as the glyoxylate cycle, MAPK signaling, and photosynthesis. (D) Metabolic and regulatory pathway model illustrating the activation of the glyoxylate cycle (e.g., MLS) and its integration with hormone signal transduction. (E) WGCNA co-expression network identifying ERF1 (Nmin_6G000717 and Nmin_12G001018) as central hub genes that integrate ethylene, jasmonate, and ABA signaling to balance rapid growth with immune defense.

Seed germination in *N. minuta* represents a critical transition from heterotrophy to autotrophy. PCA based on genome-wide expression patterns divided this process into three phases: Soaking (24–72 h), Sprout (radicle and first leaf emergence), and Seedling (7–21 days post-sowing) (Fig. 5).

Pairwise comparison revealed a massive transcriptomic shift, particularly during the transition from Soaking to Sprout, which involved 5499 DEGs. This phase marks the core ‘transcriptional ignition’ of the life cycle. *K*-means clustering categorized these dynamic genes into six expression modules that reveal the functional programming of the lifecycle: (i) Metabolic Ignition (Clusters 1 and 5, 3040 genes): Genes involved in the glyoxylate cycle (MLS, Nmin_4G001281) and purine metabolism peak during soaking and rapidly decline. This represents the ‘Metabolic Reprogramming Engine,’ which converts stored lipids into carbohydrates and energy to fuel the initial breakout of the embryo. (ii) Signal Integration (Cluster 4, 1271 genes): These genes peak during the sprout phase and are enriched in MAPK signaling and plant hormone transduction (ethylene, ABA, and JA crosstalk). (iii) Establishment (Clusters 2, 3, and 6, 3959 genes): These genes are upregulated during the seedling phase and are enriched in photosynthesis-antenna proteins and phenylpropanoid biosynthesis, marking the assembly of the photosynthetic apparatus and secondary metabolic defenses.

### ERF1 as a central hub for growth-defense trade-off

Using WGCNA, we identified a ‘blue’ module (1091 genes) consisting of positively co-expressed genes that is highly correlated (|*r*| > 0.8) with the developmental progression of the seed. Topology analysis of this module identified two ERF1 family transcription factors (Nmin_6G000717 and Nmin_12G001018) as the top hub genes, each with a connectivity degree of 37. The ERF1 hubs are tightly co-expressed with the core ABA-signaling kinase SnRK2.4 and a family of seven Pathogenesis-Related (PR1) genes, including Nmin_10G000233. This reveals an ‘ERF1-centered regulatory model’ where the same hub integrates ethylene and jasmonate signals to promote germination while simultaneously activating ABA-dependent stress responses and PR1-mediated immune barriers. This mechanism ensures that the rapid growth triggered by the glyoxylate cycle is balanced with robust protection against pathogens in the aquatic environment, a critical survival strategy for early-diverging angiosperms.

**Figure 6:**
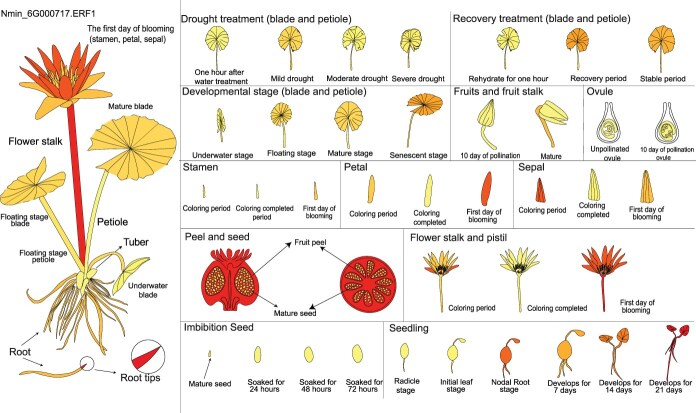
Expression patterns of Nmin_6G000717.ERF1 across tissues and treatments.

The expression profile of Nmin_6G000717.ERF1 exhibits significant spatio-temporal dynamics across the entire life cycle and environmental responses of *N. minuta*. The gene is constitutively expressed across diverse vegetative tissues, with notable activity in the root tips, tubers, and throughout heterophyllous leaf development from underwater to senescent stages. During reproductive growth, ERF1 is highly coordinated with the pigmentation and maturation of floral organs, particularly during the transition to the first day of blooming. Furthermore, the gene shows strong sensitivity to water status, with marked transcriptional shifts during progressive drought stress and subsequent physiological recovery in both blades and petioles, suggesting a pivotal role for ERF1 in mediating aquatic adaptation and abiotic stress resilience (Fig. 6).

## Discussion

### Evolutionary and technical significance of the T2T genome

The assembly of a telomere-to-telomere (T2T) genome for *N. minuta* is a watershed moment for research into the ANA-grade. Previous attempts at assembling water lily genomes, such as the initial *N. colorata* draft, provided invaluable insights into early angiosperm evolution but remained fragmented in heterochromatic regions [2]. By resolving all 14 centromeres and 27 telomeres, this study provides the first gap-free map of a basal aquatic herb, allowing for precise identification of genes previously hidden in repetitive landscapes.

The 382 Mb *N. minuta* genome is remarkably compact compared to its relative *N. colorata* (409 Mb) and significantly smaller than larger members of the family like *Victoria amazonica* (>4500 Mb) [18]. This compactness, combined with its diploid status, makes it an ideal reference for identifying ancestral gene blocks and signatures of the Nymphaealean whole-genome duplication event that occurred ~120 million years ago [19]. The absence of extensive modern polyploidy provides a ‘clean’ genomic canvas to study the regulation of flowering transitions and the parallel evolution of floral scents and colors in basal angiosperms [19].

### *Nymphaea minuta*: The emergent model for basal angiosperms

For the past decade, *N. thermarum* was championed as the premier model system for early flowering plant research due to its miniature size and short generation time [15]. Eventually, the results presented here argue strongly for the adoption of *N. minuta* as a superior laboratory model.

The technical advantages of *N. minuta* are profound. It is far easier to cultivate, tolerates diverse nutrient environments, and exhibits high seed production per flower compared to the specialized requirements and fragile seed viability of *N. thermarum*. Furthermore, the presence of vegetative tubers allows for the maintenance of clonal lines, which is invaluable for stable transformation and genetic experimentation. The ‘dimorphic’ growth habit of *N. minuta*, producing both underwater cleistogamous and emergent chasmogamous flowers, offers a unique developmental system to study phenotypic plasticity and the evolution of breeding systems in response to fluctuating water levels.

### Molecular insights into aquatic stress adaptation

The identification of the ‘Sequential Dual-Module Relay’ provides a new conceptual framework for how ancient aquatic plants navigate environmental instability. Most plants utilize a MAPK-driven transcriptional response to perceive stress and initiate protective programs [20]. While *N. minuta* shares this Phase I mechanism, its Phase II shift toward the RNA degradation pathway during severe drought is a more specialized survival strategy.

The LSM1-7 complex is a conserved eukaryotic component of the mRNA decapping machinery [21]. In *N. minuta*, the strong and specific induction of LSM1/2 and ENOC during severe dehydration suggests a systemic ‘transcriptome emergency remodeling’. Rather than trying to modulate thousands of individual growth pathways with limited cellular resources, the plant effectively clears the transcriptional slate. This mechanism likely prevents the wasteful synthesis of growth-related proteins when water is scarce, while maintaining the stability of survival-essential transcripts. This post-transcriptional control allows for the ‘growth mode’ to be efficiently switched back on as soon as moisture is restored, a strategy that is arguably essential for plants living in ephemeral pools or tidal zones.

### Coordination of energy and programming during germination

The transition from a dormant seed to an autotrophic seedling is arguably the most vulnerable phase in any plant’s life cycle [14]. In *N. minuta*, we identified an ‘Energy-Program Coordination’ model that governs this transition. The early soaking phase is dominated by the glyoxylate cycle, where genes like MLS mobilize lipid reserves to provide the metabolic foundation for morphogenesis [14].

The transition to the sprout phase is marked by the activation of the ERF1 hub. In higher plants, ERF1 is known to integrate ethylene and jasmonate signals to regulate defense and developmental trade-offs [22]. The presence of this hub at the base of the angiosperm tree suggests that the molecular logic of balancing growth and immunity is ancestral. By co-regulating the ABA pathway (via SnRK2.4) and an expanded cluster of PR1 defense genes, ERF1 acts as the master conductor, ensuring that the energy released from lipid mobilization is not just used for expansion, but for building a robust defense barrier against aquatic pathogens [23].

## Materials and methods

### Plant material and sample collection

*Nymphaea minuta* (syn. *Nymphaea dimorpha*) plants were originally sourced from Madagascar and cultivated at the Nanfan Innovation Center in Sanya, China. All plants were maintained in a greenhouse under standard conditions (14 h light/10 h dark cycle, 28–30°C). For the developmental transcriptome atlas, we collected 165 samples from 15 representative organs across three biological replicates. These included leaves (different stages), floral organs (sepal, petal, stamen, pistil, ovule), seeds (imbibition for 24, 48, 72 h, radicle stage), seedlings (7, 14, 21 days), and structural components (petiole, pedicel, fruit stalk). For dehydration treatments, mature leaves and petioles were subjected to a water-loss gradient: sampling occurred at 1 h post-removal from water, at the appearance of mild and severe wilting, and at 1 h and 24 h post-rewatering.

### Genome sequencing and T2T assembly

Genomic DNA was extracted from young leaves using an improved CTAB method and purified using Qubit and pulsed-field electrophoresis. For PacBio sequencing, DNA was sheared to 15–20 Kb using the Megaruptor 3 system. Libraries were constructed with the SMRTbell Prep Kit 3.0 and sequenced on the PacBio Revio platform, producing 7.89 million CCS reads (QV ≥ 37). Hi-C libraries were prepared through formaldehyde cross-linking, DpnII digestion, and magnetic bead capture, followed by PE150 sequencing on the MGI platform (215 million valid pairs).

The initial assembly was performed with hifiasm v0.25.0 using default parameters [24]. Chromosomes were scaffolded using Juicer and 3D-DNA pipelines [25] using 50.13 Gb Hi-C reads. Gaps were filled using TGS-GapCloser v1.2.1 [26] and single-nucleotide errors were polished with NextPolish v1.4.1 [27]. Assembly completeness was evaluated using BUSCO (embryophyta_odb12) [28] and accuracy via Merqury [29].

### Genomic landmark identification and annotation

Centromeric regions were identified using quartet_centrominer.py based on local repetitive sequence density and Hi-C contact maps. Telomeres (TTTAGGG tandem repeats) were identified at both ends of each chromosome using quartet_teloexplorer.py [30]. Repetitive elements were characterized using EDTA v2.2.2 [31] to generate a *de novo* species-specific TE library. Gene models were predicted using BRAKER3 v3.0.3 [32], integrating protein evidence from related species and RNA-seq data from this study.

### Transcriptomic data analysis

RNA was extracted using the TRIzol method and libraries were prepared with the TruSeq Stranded Total RNA Kit. Sequencing was performed on Illumina HiSeq 2500 and NovaSeq 6000 platforms. Raw reads were processed with fastp (v0.23.2) [33] and aligned to the T2T reference using HISAT2 (v2.2.1) [34]. Expression levels were estimated as TPM using StringTie (v2.1.7) [35].

Dimensionality reduction and visualization were performed using the UMAP R package. Differential expression analysis was conducted with DESeq2 v1.42.0 [36] (|log2FC| ≥ 1, FDR ≤ 0.05). Temporal expression patterns were analyzed using Mfuzz [37] (soft clustering) and K-means clustering. GO and KEGG enrichment analyses were performed using clusterProfiler v4.2.2 [38].

### Co-expression network construction

WGCNA v1.71 [39] was used to build a signed hybrid co-expression network from 9255 high-variance genes. A soft-thresholding power of 6 was applied to a signed adjacency matrix to achieve scale-free topology (*R*^2^ > 0.85). Modules were identified with a minimum size of 30 and merged at a height of 0.25. Hub genes were prioritized based on high Module Membership (kME > 0.8) and intra-modular connectivity. Networks were visualized in Cytoscape v3.9.0 [40].

### Data visualization and statistics

All statistical tests were performed in R v4.2.1 and GraphPad Prism [41]. Significance was defined as *P* < 0.05. Data are presented as mean standard error. Visualizations were generated using ggplot2 [42], pheatmap [43], and ComplexHeatmap [44].

## Conclusions

This study delivers the first gap-free, telomere-to-telomere genome assembly of *N. minuta*, establishing it as a foundational resource for the study of basal angiosperms. By resolving the centromeric and telomeric landscapes of this ancient lineage, we provide a definitive reference for comparative genomics in the Nymphaeales. Our integrated transcriptomic atlas reveals a hierarchy of regulatory mechanisms—ranging from the early MAPK signals to the late post-transcriptional RNA degradation relay—that enable leaf resilience to water fluctuations. Furthermore, we elucidate the molecular architecture of seed germination through an ERF1-centered coordination model, balancing metabolic mobilization with immune defense. These findings not only illuminate the survival strategies of one of the world’s most ancient flowering plant lineages but also identify key candidate genes and pathways for the improvement of ornamental and economic aquatic crops in a changing climate.

## Supplementary Material

Web_Material_uhag085

## Data Availability

The raw data for the genome sequencing and transcriptome sequencing could be found in NGDC (https://ngdc.cncb.ac.cn/) with project ID: PRJCA021721. Genomic assembly and annotations could be found at https://bioinformatics.hainanu.edu.cn/worp/community/download.
